# Risk prediction for cardiovascular events and all-cause mortality in maintenance hemodialysis patients

**DOI:** 10.3389/fmed.2025.1660154

**Published:** 2025-10-27

**Authors:** Mengxia Cao, Jialing Feng, Xiao Liu, Xiangqiong Wen, Santao Ou

**Affiliations:** ^1^Department of Nephrology, The Affiliated Hospital, Southwest Medical University, Luzhou, Sichuan, China; ^2^Sichuan Clinical Research Center for Nephrology, Luzhou, Sichuan, China; ^3^Metabolic Vascular Disease Key Laboratory of Sichuan Province, Luzhou, Sichuan, China; ^4^Department of Nephrology, The People's Hospital of Pudong New District in Shanghai, Shanghai, China

**Keywords:** hemodialysis, cardiovascular event, all-cause mortality, machine learning, predictive model

## Abstract

**Objective:**

This study is designed to develop predictive models for cardiovascular events (CVE) and all-cause mortality in maintenance hemodialysis (MHD) patients using machine learning (ML) algorithms. Furthermore, we aim to compare the performance of these ML-based models with that of traditional Cox regression models.

**Methods:**

We conducted a retrospective study that included 275 patients who underwent MHD treatment from January 1, 2020, to January 1, 2022. We collected comprehensive data on their demographic characteristics, comorbidities, medication history, and baseline laboratory values, and followed up with them throughout the study period. To develop predictive models for CVE and all-cause mortality, we employed several ML algorithms, including Logistic Regression (LR), Support Vector Machine (SVM), Random Forest (RF), Decision Tree (DT), Extreme Gradient Boosting (XGBoost), and Naive Bayes Model (NBM). Finally, we compared the predictive accuracy of the ML models with that of Cox regression models by evaluating their respective AUC values.

**Results:**

During a median follow-up period of 50.0 months, 119 patients experienced CVE and 75 patients died. The XGBoost model emerged as the most accurate predictor of CVE. The AUC values for predicting CVE at 1, 2, 3, and 4 years were 0.650, 0.702, 0.742, and 0.755 respectively. The accuracy, F1 score, recall, and precision were 0.731, 0.694, 0.706, and 0.683. Key predictors identified included a history of cardiovascular disease, total iron-binding capacity, body mass index, red blood cell count, mean corpuscular hemoglobin, and serum magnesium levels. For predicting all-cause mortality, the RF model demonstrated the highest performance. The AUC values for predicting all-cause mortality at 1, 2, 3, and 4 years were 0.903, 0.931, 0.882, and 0.862 respectively; the accuracy, F1 score, recall, and precision were 0.796, 0.517, 0.400, and 0.732. Significant predictors included dialysis vintage, post-dialysis β2-microglobulin levels, B-Carboxy-Terminal Peptide of Type I Collagen, total bilirubin, lymphocyte count, lactate dehydrogenase, mean corpuscular hemoglobin concentration, and the use of roxadustat. Across all endpoints, the ML models demonstrated better discrimination than Cox regression models.

**Conclusions:**

Overall, ML models provided a more reliable prognostic assessment than Cox regression models for predicting CVE and all-cause mortality in MHD patients over the observation period.

## Introduction

Cardiovascular disease (CVD) is the predominant cause of morbidity and mortality among patients undergoing maintenance hemodialysis (MHD), contributing to a substantial proportion of adverse outcomes in this high-risk population (1–3). The pathogenesis of CVD in this context is multifaceted, involving a complex interplay of both traditional and non-traditional risk factors (4–6). Chronic kidney disease (CKD) patients are at high risk and burden of CVD and cardiovascular death, which increases in a continuous fashion with worsening renal function (7–9). Traditional cardiovascular risk factors, including hypertension, dyslipidemia diabetes mellitus and advanced age, have an important role in the progression of CVD in patients who have a decreased glomerular filtration rate, in particular in those with mild-to-moderate CKD patients (1, 10–12). Unfortunately, many patients miss the optimal window for intervention, often leading to delayed treatment initiation.

However, traditional CVD risk stratification tools, such as the Framingham Risk Score, Systematic Coronary Risk Evaluation, and Atherosclerotic Cardiovascular Disease Risk Estimator, often fall short in accurately predicting CVD risk among MHD patients (13). This limitation is partly due to the fact that these models primarily incorporate traditional cardiovascular and cerebrovascular risk factors, while largely neglecting the unique contributions of chronic kidney disease (CKD) and the dialysis process itself. As a result, they may significantly underestimate the true CVD risk in this population. The Cox proportional hazards model has long been the standard for survival analysis and risk prediction in clinical research. However, it is not without limitations (13). It assumes a linear relationship between covariates and risk, as well as independence among covariates. Moreover, it struggles to effectively screen and integrate large volumes of high-dimensional data. Given these challenges, there is an urgent need for a clinical prognostic assessment tool that offers highly reliable predictive capability specifically tailored for MHD patients.

In recent years, the rapid advancement of artificial intelligence (AI) technology has ushered in a new era of possibilities within the medical field (14–16). Machine learning (ML), a key subset of AI, has emerged as a powerful tool that automates decision-making processes by learning from data through the development and training of sophisticated algorithms (17–20). Over the past few years, ML has been increasingly utilized to construct clinical prediction models, demonstrating remarkable potential in enhancing diagnostic and prognostic accuracy. In many clinical scenarios, these models have outperformed traditional statistical methods, highlighting their superior ability to capture complex relationships within data (21–23).

Most patients with end-stage renal disease (ESRD) undergo MHD therapy, typically three times per week. This frequent treatment schedule generates a wealth of clinical data, including hospitalization records, medication use, adverse events, and laboratory test results. Motivated by recent advancements, this study aims to develop predictive models for cardiovascular events (CVE) and all-cause mortality in MHD patients using ML algorithms. These models will be compared with traditional Cox regression models, with the goal of providing a more accurate tool for risk stratification in this high-risk population.

## Materials and methods

### Study population

This study enrolled patients who had MHD treatment for at least 3 months and were aged 18 years or older at the hemodialysis unit of the Affiliated Hospital of Southwest Medical University between January 1, 2020, and January 1, 2022. The exclusion criteria encompassed the following conditions: a history of peritoneal dialysis or renal transplantation; underlying malignancy; severe infection; severe hepatic insufficiency; or active tuberculosis. Additional exclusions included multiple myeloma, bone tumors, and other disorders affecting calcium and phosphorus metabolism *in vivo*; parathyroidectomy; and an active phase of autoimmune disease requiring high-dose glucocorticoids or immunosuppressive therapy. This study was approved by the Ethics Committee of the Affiliated Hospital of Southwest Medical University (Approval No.: KY2024300), as an exempt study with a waiver of informed consent, permitting a retrospective review of medical records.

### Data set

The study employed a comprehensive dataset comprising four key components: demographic characteristics (9 variables), comorbidities (6 variables), medication history (9 variables), and baseline laboratory values (63 variables). All predictor variables were extracted from electronic medical records, with specific details provided in Supplementary Table 1.

### The endpoints

The endpoints of this study were the first occurrence or recurrence of CVE and all-cause mortality. A broad definition of CVE was adopted (24), which included stroke (including transient ischemic attacks), severe cardiac arrhythmias (such as ventricular fibrillation, ventricular tachycardia, atrial fibrillation, atrial flutter, severe bradycardia, and heart block), acute myocardial infarction, unstable angina pectoris, coronary artery revascularization, development of various types of heart failure (HF) requiring hospitalization, sudden cardiac death, and peripheral vascular disease necessitating intervention or amputation. The follow-up period concluded on June 30, 2024.

### Statistical analysis

Data were stored and managed using Excel 2016, while statistical analyses were conducted using the R language (version 4.4.1). Variables with missing rates exceeding 30% were excluded from the analysis. For variables with missing rates ≤ 30%, the following imputation methods were employed: median imputation, mean imputation, or mode imputation, depending on the variable's trend. For count data, random interpolation was performed based on the proportion of available positive and negative data. Normality tests were conducted on continuous variables. Normally distributed variables were presented as mean ± standard deviation, while non-normally distributed variables were presented as median (P25, P75). Categorical variables were expressed as proportions. Comparisons of variable distributions between groups were performed using ANOVA or the Kruskal–Wallis *H* test, as appropriate. All statistical tests were two-sided, with a significance level set at *P* < 0.05.

### Development of the Cox model

The Cox regression model was constructed using the “coxph” function in R. Initially, univariate Cox regression analyses were conducted to identify potential risk factors associated with CVE and all-cause mortality among MHD patients, with significance set at *P* < 0.05. Variables that were significant in the univariate analyses were subsequently included in the multivariate Cox regression model to determine independent predictors. The stability of the model was evaluated using 5-fold cross-validation. For each fold, risk scores were calculated, and the validation sets along with their predicted outcomes were integrated. The optimal cutoff value for risk stratification was determined using the ‘surv_cutpoint‘ function. Kaplan-Meier survival curves were generated using the ‘ggsurvplot‘ function, and a nomogram was created with the ‘nomogram‘ function. The predictive performance of the model was assessed by plotting time-dependent receiver operating characteristic (ROC) curves, which integrate specificity and sensitivity. A model with an area under the curve (AUC) greater than 0.70 was considered to have good discrimination.

## Development of the ML model

### Feature selection

This step aims to identify a subset of features from the original dataset that maximizes the outcome benefit, thereby reducing model complexity and enhancing generalizability. For feature selection, we employed the Sequential Feature Selector (SFS) method in conjunction with a Random Forest regressor. SFS is a greedy algorithm that iteratively adds or removes features to optimize model performance. The model-building process began with an empty feature set, and features were added incrementally in steps of 2. This iterative process continued until either a predefined number of features was reached or further improvements in model performance plateaued.

### Model development

ML models were developed using Python software (version 3.10.0). Six classical ML algorithms were employed to predict the risk of CVE and all-cause mortality in MHD patients. These algorithms included logistic regression (LR), support vector machine (SVM), random forest (RF), decision tree (DT), extreme gradient boosting (XGBoost), and Naive Bayesian model (NBM). The primary functions of these algorithms were defined, and the models were iteratively trained with varying numbers of features to generate corresponding prediction results and performance reports. Five-fold cross-validation was used for internal validation, and the average values of these validations were accepted as the final results to mitigate the risk of overfitting.

### Model evaluation

As our ML models were binary classifiers, their performance was evaluated using several key metrics: accuracy, recall, precision, F1-score, and AUC. These metrics were derived from the four possible outcomes of binary classification: true positive (TP), true negative (TN), false positive (FP), and false negative (FN) (Table 1). Accuracy measures the proportion of correct predictions (both TP and TN) among all subjects. Recall (also known as sensitivity or the “TP rate”) represents the proportion of actual non-surviving patients that are correctly identified as non-surviving by the classifier. Precision indicates the proportion of TP results among all positive predictions, reflecting the classifier's ability to avoid FP results. F1-score is the harmonic mean of precision and recall, providing a balanced measure of the two. Specificity (also known as the “TN rate”) measures the proportion of actual surviving patients that are correctly predicted to survive. AUC was computed by plotting sensitivity against 1-specificity across all possible cutoff points. It serves as an overall measure of the model's discrimination ability, with higher AUC values indicating better performance.

**Table 1:** Confusion matrix for machine learning classification criteria.

**Actual/predicted**	**Positive**	**Negative**
Positive	Ture positive (TP)	False negative (FN)
Negative	False positive (FP)	Ture negative (TN)

$Accuracy=\frac{TP+TN}{TP+TN+FP+FN}$

$Precision=\frac{TP}{TP+FP}\;$

$Recall=\frac{TP}{TP+FN}\;$

$F1-score=\frac{2\times Precision\times Recall}{TP+FP}\;$

### Interpretability

To enhance the interpretability of ML models, Shapley Additive exPlanations (SHAP) were employed. SHAP leverages the concept of SHAP values, which are grounded in game theory, to quantify the importance of each feature in the model. By calculating the SHAP value for each feature, it assesses the contribution of that feature to the prediction outcome. This approach generates both visual and quantitative interpretations, enabling users to understand the decision-making process of the ML model more transparently and thereby enhancing the model's credibility.

## Results

### Baseline characteristics

**Figure 1:**
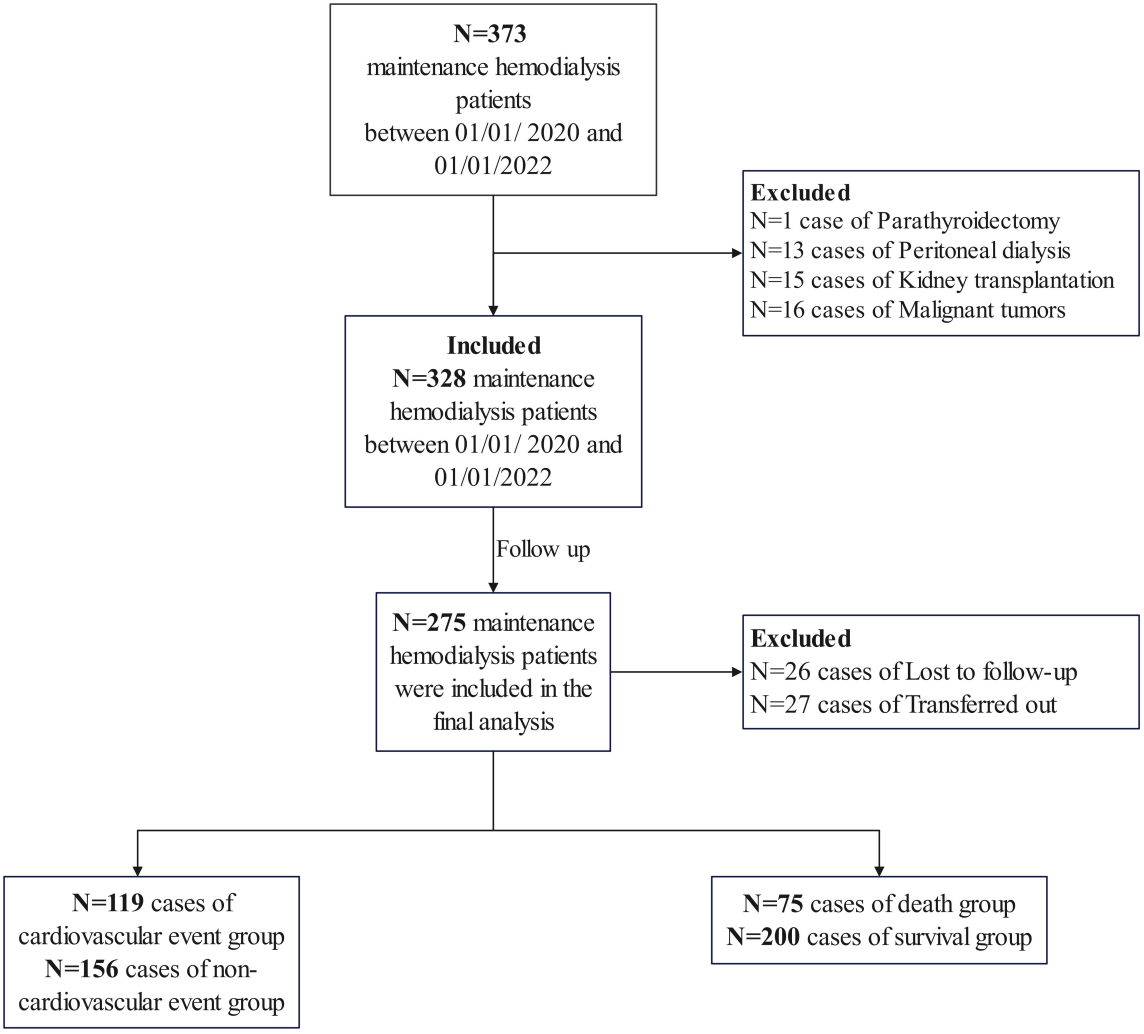
The flowchart of patient selection for this study.

The flowchart of patient selection for this study is presented in Figure 1. A total of 373 patients undergoing MHD were identified at our hospital between January 1, 2020, and January 1, 2022. Patients with a history of parathyroidectomy (*n* = 1), peritoneal dialysis (*n* = 13), renal transplantation (*n* = 15), or malignancy (*n* = 16) were excluded. Ultimately, 328 patients were included in the study and followed up until June 30, 2024. During this period, 26 patients were lost to follow-up, and 27 were transferred to other dialysis centers. Consequently, 275 participants were included in the final analysis. The median age of the participants was 56.0 years [interquartile range (IQR) 48.0–67.0], and the median dialysis vintage was 64.0 months (IQR 41.0–92.0). The cohort comprised 62.2% males. The primary underlying renal diseases were chronic glomerulonephritis (39.3%), diabetic nephropathy (28.7%), and hypertensive nephropathy (18.9%). The remaining 13.1% of patients had other renal diseases, including polycystic kidney disease, obstructive nephropathy, gouty nephropathy, etc. The overall rate of missing data was 0.51%, and these missing values were imputed using the median method, as detailed in Supplementary Table 1.

### Follow-up outcomes of CVE

The median follow-up period was 50.0 months (IQR 34.5–53.0). During this period, a total of 119 patients (43.3%) experienced CVE. Among these patients, 80 were men (incidence rate of 67.2%) and 39 were women (incidence rate of 32.8%). The specific types of CVE included: HF in 49 cases (41.18%), cerebral hemorrhage in 27 cases (22.69%), cerebral infarction in 13 cases (10.92%), unstable angina pectoris in 11 cases (9.24%), cardiac arrhythmia in 9 cases (7.56%), myocardial infarction in 4 cases (3.36%), peripheral vascular disease in 4 cases (3.36%), and transient cerebral ischemic attack in 2 cases (1.68%). Baseline characteristics were compared between the CVE group and the non-CVE group, with detailed data presented in Supplementary Table 2.

### Follow-up outcomes of all-cause mortality

During the follow-up period, a total of 75 patients (27.3%) died. Of these, 52 (69.3%) were male and 23 (30.7%) were female. CVE were the cause of death in 42 patients (56%). The remaining 33 patients (44%) died from non-CVE causes, including respiratory failure, sepsis, poisoning, gastrointestinal hemorrhage, uremic encephalopathy, suicide, and an unknown cause. Baseline characteristics were compared between the death and survival groups, with detailed data presented in Supplementary Table 3.

### Cox model prediction results

**Figure 2:**
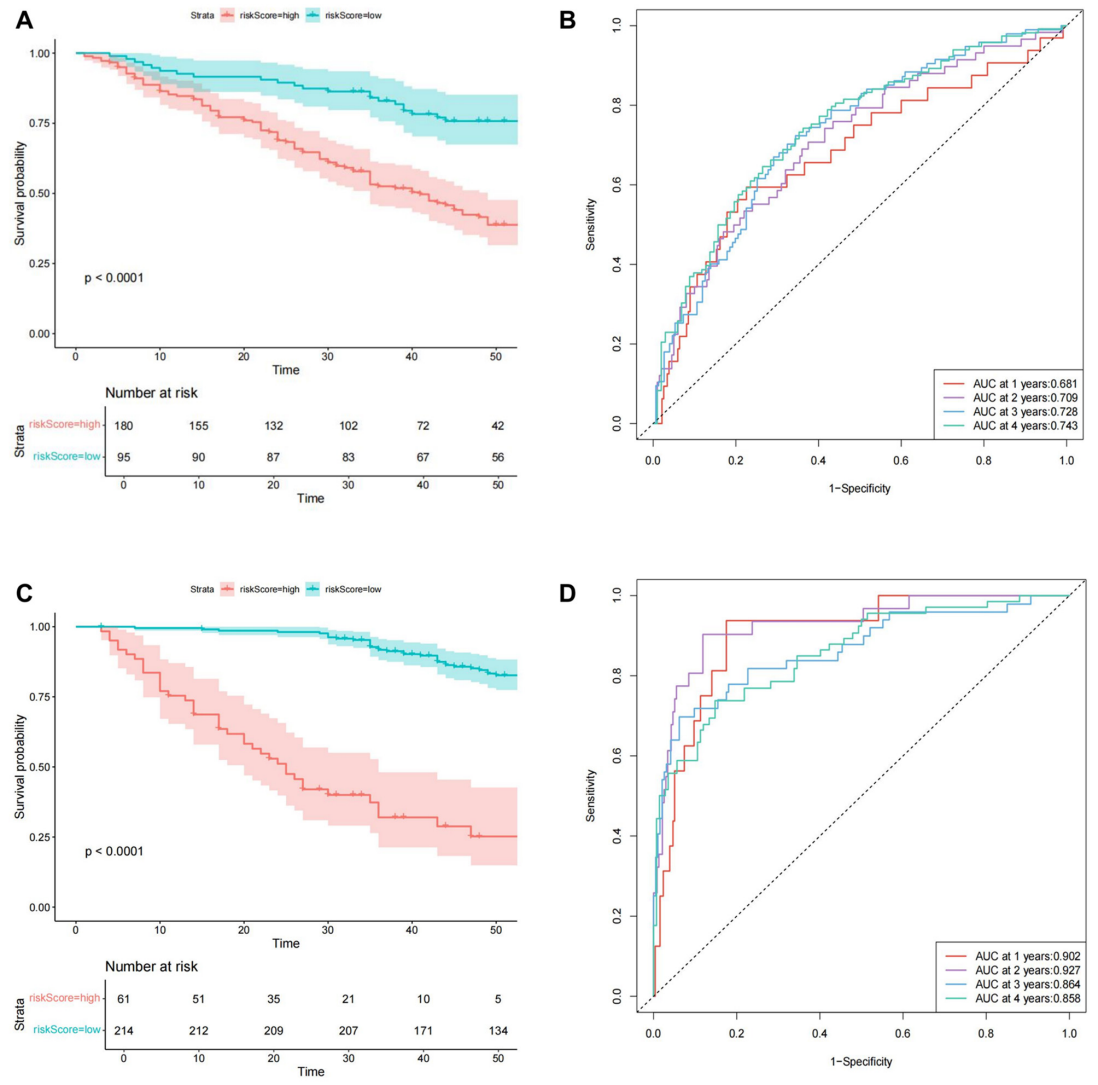
The Cox model prediction results. **(A)** The Kaplan-Meier survival plot of cardiovascular events. **(B)** The time-dependent ROC plot of cardiovascular events. **(C)** The Kaplan-Meier survival plot of all-cause mortality. **(D)** The time-dependent ROC plot of all-cause mortality.

In predicting CVE, multivariate Cox regression analysis identified several independent risk factors: a history of CVD [hazard ratio (HR): 1.984, 95% confidence interval (CI): 1.282–3.070], creatine kinase isoenzyme (CK-MB) (HR: 1.098, 95% CI: 1.001–1.204), red cell distribution width-coefficient of variation (RDW-CV) (HR: 1.007, 95% CI: 1.001–1.012), and mean corpuscular hemoglobin (MCH) (HR: 0.935, 95% CI: 0.875–0.998) (*P* < 0.05 for all). Based on a cutoff value of 0.61, subjects were stratified into high-risk (cutoff > 0.61) and low-risk (cutoff ≤ 0.61) groups for CVE, comprising 180 and 95 cases, respectively. The Kaplan-Meier survival plot demonstrated a significant difference in survival between the high-risk and low-risk groups (*P* < 0.001) (Figure 2A). The time-dependent ROC plot showed AUC values for predicting CVE at 1, 2, 3, and 4 years were 0.681, 0.709, 0.728, and 0.743, respectively (Figure 2B).

**Figure 3:**
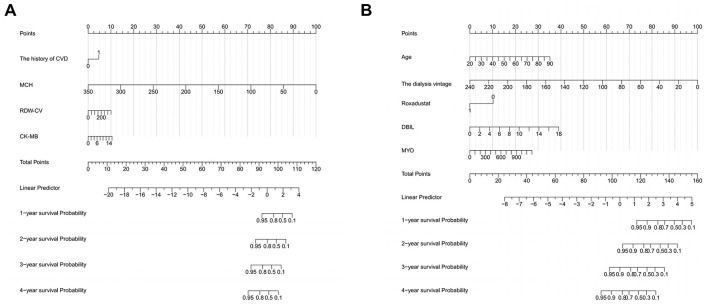
Nomograms for predicting cardiovascular events **(A)** and all-cause mortality **(B)**. CVD, cardiovascular disease; MCH, mean corpuscular hemoglobin; RDW-CV, red cell distribution width-coefficient of variation; CK-MB, creatine kinase isoenzyme; DBIL, direct bilirubin; MYO, myohemoglobin.

In predicting all-cause mortality, multivariate Cox regression analysis identified several independent risk factors: age (HR: 1.030, 95% CI: 1.003–1.058), direct bilirubin (DBIL) (HR: 1.235, 95% CI: 1.003–1.520), myohemoglobin (MYO) (HR: 1.0023, 95% CI: 1.000–1.004), dialysis vintage (HR: 0.95, 95% CI: 0.935–0.966), and the use of roxadustat (HR: 0.395, 95% CI: 0.193–0.810). Based on a cutoff value of 1.87, subjects were stratified into high-risk (cutoff > 1.87) and low-risk (cutoff ≤ 1.87) groups for all-cause mortality, comprising 61 and 214 cases, respectively. The Kaplan-Meier survival plot demonstrated a significant difference in survival between the high-risk and low-risk groups (*P* < 0.001) (Figure 2C). The time-dependent ROC plot showed AUC values for predicting all-cause mortality at 1, 2, 3, and 4 years were 0.902, 0.927, 0.864, and 0.858, respectively (Figure 2D). Finally, the nomograms for predicting CVE (Figure 3A) and all-cause mortality (Figure 3B) were constructed based on the selected independent factors. Each variable was first scored on its corresponding subscale. Subsequently, the scores of all variables were summed to obtain a total score, which corresponded to the risk of CVE or all-cause mortality occurrence.

### ML model prediction results

**Figure 4:**
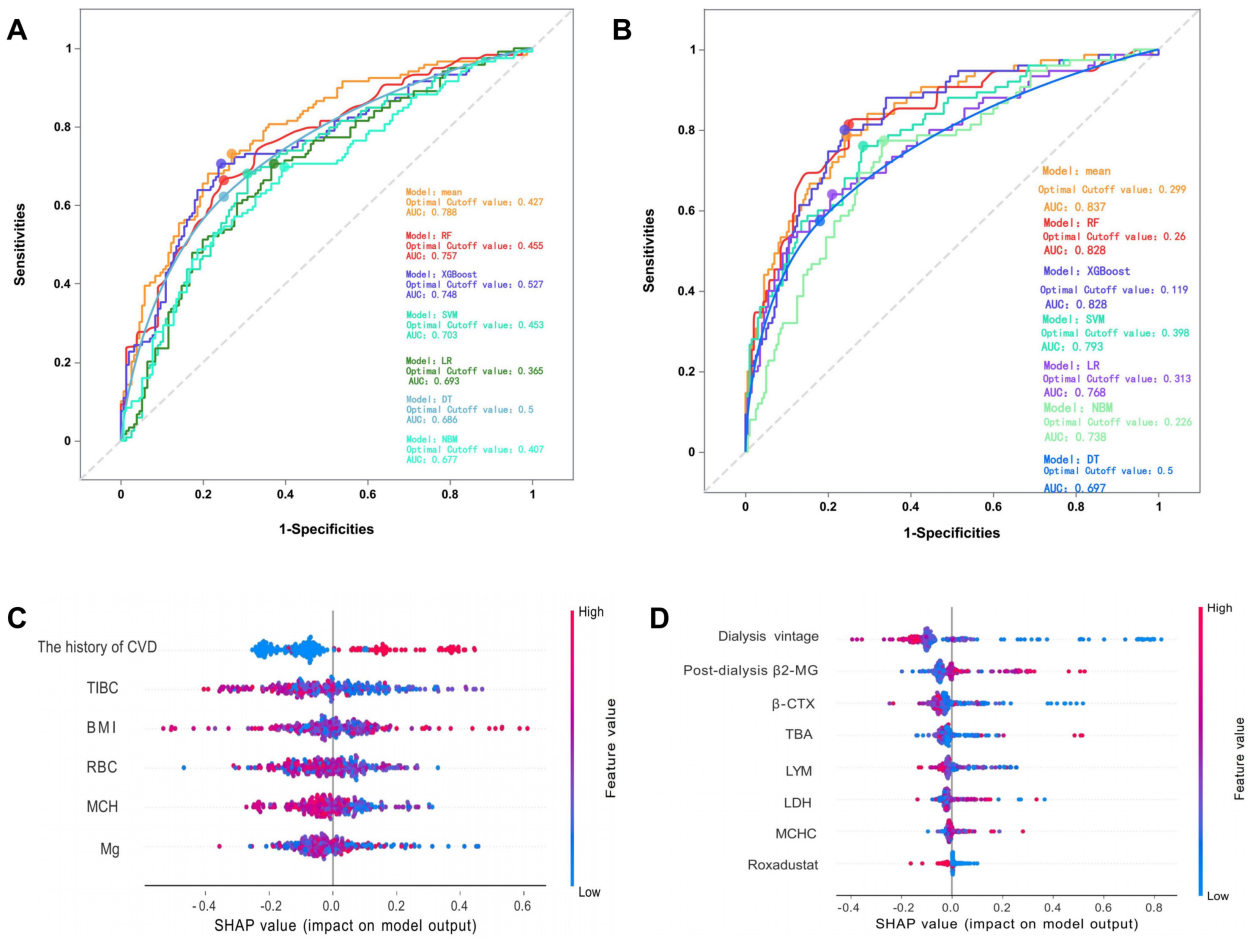
**(A)** The overall ROC curves for predicting cardiovascular events by various models. **(B)** The overall ROC curves for predicting all-cause mortality by various models. **(C)** The SHAP values of the best features in XGBoost. **(D)** The SHAP values of the best features in RF. The SHAP plot displays features in descending order of importance from top to bottom, with those at the top exerting a greater overall influence on the model's output, while those below exert a lesser influence. The horizontal axis represents the SHAP value, indicating each feature's contribution to the model's prediction outcome and its direction. A positive value signifies that the feature increases the predicted value of the model's output, while a negative value indicates that it decreases the predicted value. SHAP, SHapley Additive exPlanation; CVD, cardiovascular disease; TIBC, total iron binding capacity; BMI, body mass index; RBC, red blood cell count; MCH, mean corpuscular hemoglobin; Mg, magnesium. β2-MG, β2-microglobulin; β-CTX, B-Carboxy-Terminal Peptide Of Type I Collagen; TBA, total bilirubin; LYM, lymphocytes; LDH, lactate dehydrogenase; MCHC, mean corpuscular hemoglobin concentration.

**Figure 5:**
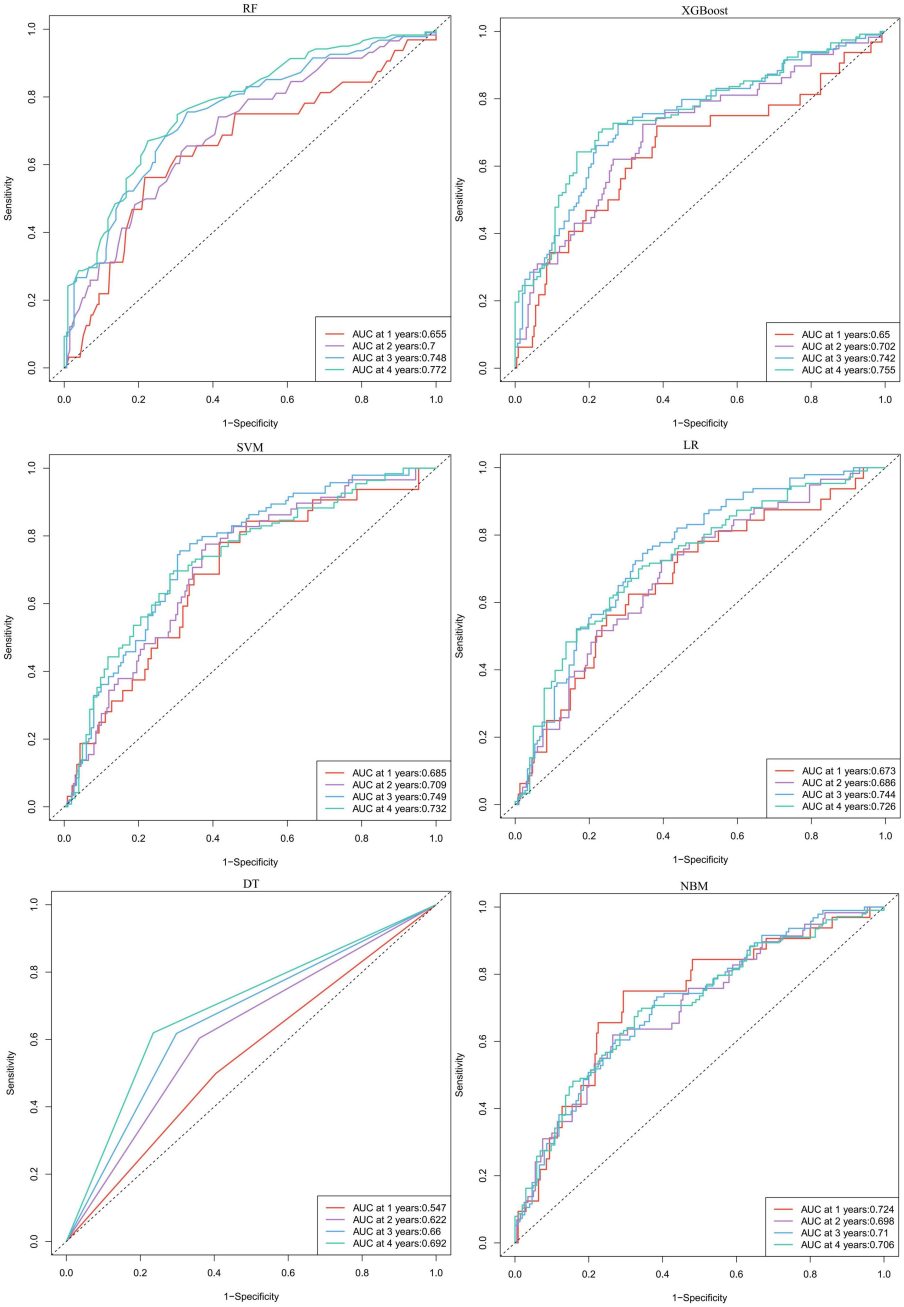
The time-dependent ROC curves for predicting cardiovascular events by six machine learning models. RF, random forest; XGBoost, extreme gradient boosting; LR, logistic regression; SVM, support vector machine; DT, decision tree; NBM, naive bayesian model.

In predicting CVE, the average overall AUC of the six ML models was 0.788. Among these models, RF achieved the highest AUC value of 0.757, followed by XGBoost, SVM, LR, DT, and NBM (Figure 4A). The time-dependent ROC curves of these models demonstrate that prediction performance improves gradually as the prediction time extends (Figure 5). Detailed performance evaluations of the six ML models are presented in Table 2. Notably, both RF and XGBoost required the fewest predictors to achieve optimal prediction performance. Although RF had the highest AUC value, XGBoost outperformed it in terms of accuracy, recall, precision, and F1 score. Therefore, XGBoost was identified as the optimal prediction model for CVE. The SHAP plot ranked the variables according to their contribution to the XGBoost model's output. The most important feature variable was the history of CVD, followed by total iron-binding capacity (TIBC), body mass index (BMI), red blood cell (RBC) count, MCH, and serum magnesium (Mg) (Figure 4C).

**Table 2:** Comparison of evaluation metrics for six machine learning models of cardiovascular events.

**Model**	**The number of optimal features**	**AUC**	**Accuracy**	**F1-score**	**Recall**	**Precision**
RF	6	0.757	0.698	0.621	0.571	0.680
XGBoost	6	0.748	0.731	0.694	0.706	0.683
Tree	14	0.686	0.695	0.638	0.622	0.655
LR	38	0.693	0.669	0.565	0.496	0.656
SVM	38	0.703	0.669	0.581	0.529	0.643
NBM	42	0.677	0.665	0.574	0.521	0.639

**Figure 6:**
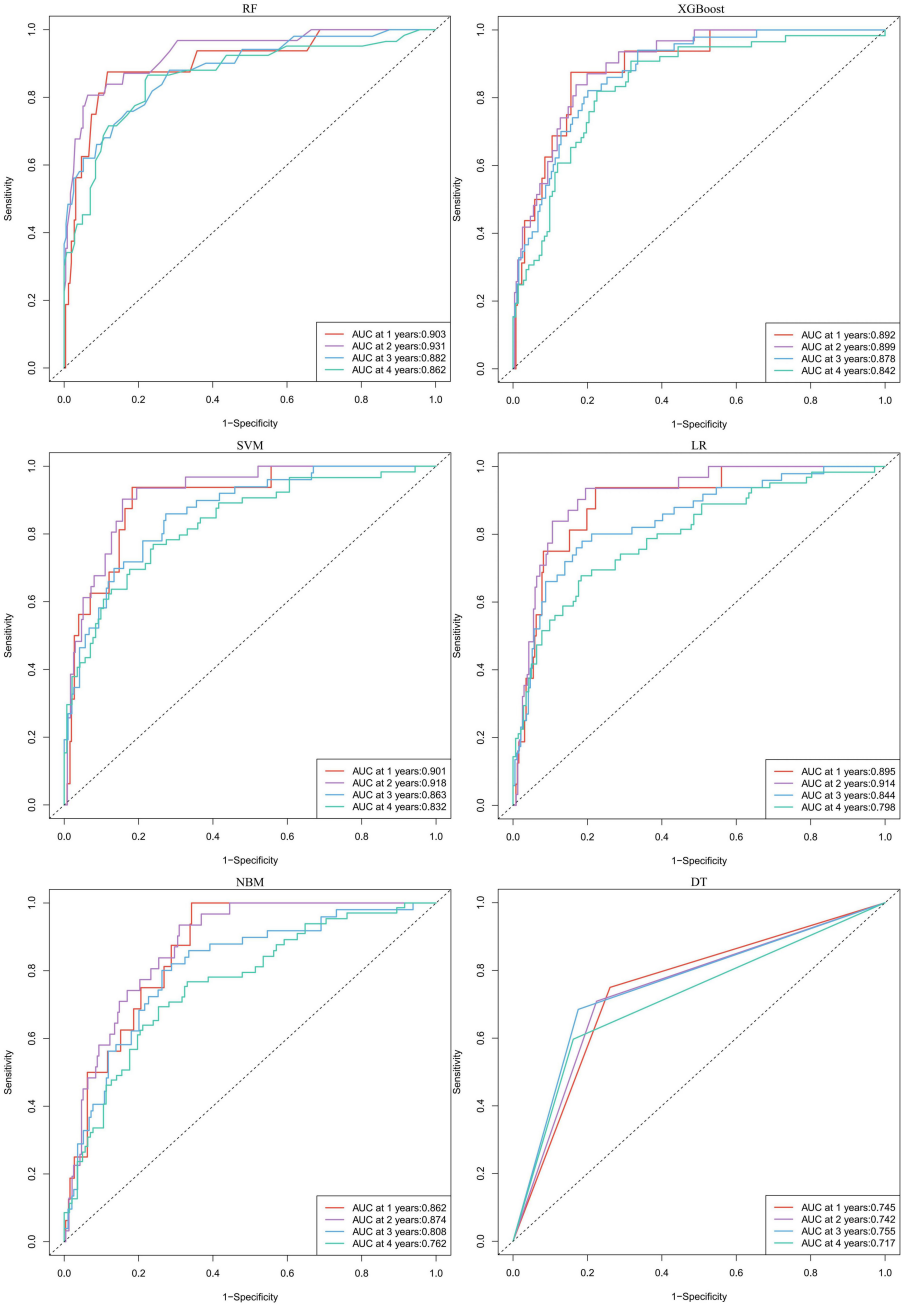
The time-dependent ROC curves for predicting all-cause mortality by six machine learning models. RF, random forest; XGBoost, extreme gradient boosting; LR, logistic regression; SVM, support vector machine; DT, decision tree; NBM, naive bayesian model.

In predicting all-cause mortality, the average overall AUC of the six ML models was 0.837. Among these models, RF and XGBoost achieved the highest AUC value of 0.828, followed by SVM, LR, NBM, and DT (Figure 4B). The time-dependent ROC curves indicate that the models exhibited the greatest superiority in predicting all-cause mortality at 2 years. However, as the prediction time extended beyond this period, the prediction performance decreased somewhat (Figure 6). As shown in Table 3, RF required the fewest predictors and demonstrated the best performance in terms of accuracy, precision, and AUC value. Therefore, RF was determined to be the best prediction model for all-cause mortality. The SHAP plot reveals that dialysis vintage is the most significant feature, followed by post-dialysis β2-microglobulin (β2-MG), B-Carboxy-Terminal Peptide of Type I Collagen (β-CTX), total bilirubin (TBA), lymphocytes (LYM), lactate dehydrogenase (LDH), mean corpuscular hemoglobin concentration (MCHC), and the use of roxadustat (Figure 4D).

**Table 3:** Comparison of evaluation metrics for six machine learning models of all-cause mortality.

**Model**	**The number of optimal features**	**AUC**	**Accuracy**	**F1-score**	**Recall**	**Precision**
RF	8	0.828	0.796	0.517	0.400	0.732
Tree	16	0.697	0.753	0.558	0.573	0.544
LR	24	0.768	0.789	0.508	0.400	0.698
SVM	24	0.793	0.775	0.500	0.413	0.633
XGBoost	26	0.828	0.793	0.571	0.507	0.655
NBM	84	0.738	0.738	0.438	0.373	0.528

### Comparison of predictive performance between Cox and ML model

As illustrated in Tables 4, 5, the XGBoost model demonstrates superior overall predictive performance compared to the Cox regression model in predicting CVE. While the Cox regression model exhibits slightly better performance in the first and second years, it still falls short of XGBoost in predicting the third and fourth years. In predicting all-cause mortality, RF consistently outperforms the Cox regression model both in overall performance and at each individual time point.

**Table 4:** Comparison of the predictive ability for cardiovascular events between XGBoost and Cox model.

**AUC**	**Overall**	**1-year**	**2-year**	**3-year**	**4-year**
XGBoost	0.748	0.650	0.702	0.742	0.755
Cox model	0.669	0.681	0.709	0.728	0.743

**Table 5:** Comparison of the predictive ability for all-cause mortality between RF and Cox model.

**AUC**	**Overall**	**1-year**	**2-year**	**3-year**	**4-year**
RF	0.828	0.903	0.931	0.882	0.862
Cox model	0.779	0.902	0.927	0.864	0.858

## Discussion

This study compared the predictive abilities of traditional Cox regression analysis and ML methods for CVE and all-cause mortality risk in MHD patients. The results indicated that ML models outperformed the Cox regression models. While ML models did not show significant advantages at certain time endpoints, this may be attributed to the relatively small sample size.

In recent years, ML algorithms have emerged as powerful tools for predictive modeling across various fields of medicine (25–27). Traditional survival analysis methods, such as Cox proportional hazards regression and logistic regression, rely on the assumption that the relationship between variables and outcomes is linear. By contrast, ML algorithms do not depend on such assumptions. They have more flexible requirements regarding data distribution and can select from a wide range of algorithms based on the characteristics of the data. Additionally, ML algorithms can train on multiple randomly selected samples and balance sample errors effectively. This ML-based approach is particularly adept at handling large, multidimensional datasets. It does not require the data to be normally distributed and mitigates the risk of overfitting through techniques such as cross-validation and regularization.

Several previous studies have compared the predictive performance of ML models with that of traditional regression models. For instance, Wang et al. (28) developed a HF prediction model using the XGBoost algorithm. The XGBoost model demonstrated significant advantages over traditional linear logistic regression in terms of accuracy (78.5% vs. 74.8%), sensitivity (79.6% vs. 75.6%), specificity (78.1% vs. 74.4%), and AUC (0.814 vs. 0.722). Similarly, Xu et al. (29) trained models using XGBoost, RF, and AdaBoost to assess the risk of 1-year and 5-year HF hospitalization and mortality in peritoneal dialysis patients, compared them with Cox regression. The results showed that the RF model (AUC = 0.853) was the best for predicting HF, and the XGBoost model (AUC = 0.871) was the best for predicting mortality, both outperforming the Cox regression models. These studies underscore the advantages of ML models in clinical risk prediction. In the context of hemodialysis patients, although Akbilgic et al. (30) and Sheng et al. (31) also employed ML methods to predict mortality risk, their studies focused on short-term risk assessment and lacked direct comparison with traditional models.

Each of the six ML models employed in this study possesses distinct characteristics. In predicting CVE, XGBoost emerged as a standout model among the six, achieving the highest accuracy, precision, and recall, with an AUC value second only to RF. Compared to other ML algorithms, XGBoost demonstrates robustness against overfitting in unbalanced datasets and can be effectively tuned for such datasets (32). The SHAP analysis revealed that history of CVD is the most contributive feature, thereby confirming its significant role in risk prediction. This finding also underscores the reliability of the ML model we constructed. CVD and CKD can be causative of each other, forming a vicious cycle. This bidirectional interaction is a characteristic of what is commonly known as the “cardiorenal syndrome.” They often share common pathophysiological mechanisms, such as oxidative stress and inflammatory responses, activation of renin-angiotensin system, abnormal signaling pathways (such as the Wnt/β-catenin signaling pathway and the TGF-β1/Smad signaling pathway), endothelial dysfunction, and vascular calcification (33). CKD patients undergoing dialysis face a higher cardiovascular risk. Each hemodialysis treatment causes drastic changes in the patient's electrolytes and hemodynamics, which can trigger subendocardial ischemia, left ventricular hypertrophy, diastolic dysfunction, and severe arrhythmias. This significantly increases the risk of acute ischemic syndrome, arrhythmias, and sudden cardiac death. A meta-analysis conducted in 2019 explored cardiovascular outcomes in MHD patients, highlighting the very high incidence of CVE, particularly among those with a history of CVD, as well as its association with increased risks of all-cause mortality and cardiac mortality (34). This suggests that heightened attention should be given to this patient subgroup to prevent the recurrence of CVE. Meanwhile, TIBC, BMI, RBC, MCH, and Mg were identified as the optimal features by the XGBoost model. Previous studies (35–38) have also associated these variables with CVD and all-cause mortality in MHD patients. Therefore, they should be considered important indicators for clinical monitoring and management.

RF is a classical and highly versatile supervised learning algorithm. It integrates multiple unrelated decision trees to construct a robust ensemble model, capable of performing both regression and classification tasks in a stochastic manner (39). Relative to traditional regression models, RF demonstrates superior capability in managing non-linear relationships and intricate interactions among variables. In our study, RF effectively predicted all-cause mortality utilizing merely eight feature variables. It attained the highest performance in terms of AUC value, accuracy, and precision. However, its recall was comparatively lower. In the SHAP plot, dialysis vintage emerged as the most significant feature. Similarly, in the RF model developed by Chen et al. (40), dialysis vintage was identified as the most influential factor in CKD progression, outweighing other factors. However, in our study, dialysis vintage negatively impacted the RF model's output. The Cox regression results also indicated that patients with shorter dialysis vintage have a relatively lower risk of all-cause mortality. From a theoretical and clinical perspective, however, patients with a longer dialysis vintage typically accumulate more cardiovascular risk factors and comorbidities. As they age, their physical function declines, placing them at higher risk for CVD and mortality. Current research suggests that the dialysis vintage is associated with an increased risk of death in HD patients and has different impacts on specific causes of mortality (41). The findings in our study may be attributed to treating dialysis vintage as a continuous variable and the relatively small sample size, which could introduce result bias. Further investigation is warranted to elucidate these findings. Additionally, post-dialysis β2-MG, β-CTX, TBA, LYM, LDH, MCHC were identified as the optimal features in RF model, indicating that these variables may play a significant role in assessing disease prognosis (42–45).

To conclude, our study aimed to accurately predict the risks of CVE and all-cause mortality in MHD patients using ML tools. Our ML prediction models exhibit several unique characteristics: Firstly, ML has demonstrated its strengths in processing large-scale medical data, making it particularly suitable for studying MHD patients with complex comorbidities. In this study, we constructed several ML models that outperformed traditional Cox regression models. Unlike Cox regression, ML models do not rely on linear assumptions, can automatically select feature variables, and provide more accurate predictions. Secondly, data noise and missing data are inevitable in real-world data collection, especially in retrospective studies. ML algorithms are well-equipped to handle these complex issues effectively. Thirdly, we employed the SHAP algorithm to interpret the ML models. This approach allows developers and users to better understand the intrinsic reasons behind the model's validity, reducing the “black box” effect and enhancing the reliability of big data analytics (46). In actual clinical practice, it is hoped that the model will be embedded into the hospital electronic medical record system as a clinical decision support tool. Risk stratification thresholds are defined based on the optimal cutoff values derived from ROC analysis, and early intervention (such as prioritized cardiology consultations and adjustment of dialysis strategies) is carried out in combination with clinical pathways. Measures such as regular model updates, user training, and clinical feedback mechanisms are adopted to reduce the risk of misclassification. In the future, ML models can transition from “research tools” to “clinical assistants,” providing personalized, interpretable, and sustainable risk management services for MHD patients. Although we concluded that demographic characteristics (9 variables), comorbidities (6 variables), medication history (9 variables), and baseline laboratory values (63 variables)-based on machine learning models provided a prognosis for predicting cardiovascular events and all-cause mortality in patients with undergoing maintenance hemodialysis, the molecular mechanism is unclear. Recent publications have shown that many risk factors, such as hypertension, renin-angiotensin system activation, and cardiorenal injury were implicated in CVD and CKD including hemodialysis (47–49). In addition, many researches have demonstrated that abnormal hyperlipidemia and inflammation play a significant role in CVD and CKD (50–53). Moreover, a large amount of literature has shown that the imbalance of intestinal flora and its metabolites is involved in CVD and CKD (54–58).

The current study also has several limitations. First, our data were derived from a single center with a relatively small sample size, which may limit the generalizability of our findings. Second, although our prediction model demonstrated strong performance, it has not yet undergone external validation. Further research is needed to confirm its clinical applicability. Third, this study utilized only baseline data from MHD patients and was unable to assess the impact of potential fluctuations in these variables on CVE and all-cause mortality over time. Future research should focus on conducting larger-scale, multicenter studies and performing external validation to further verify and optimize the model. Additionally, incorporating longitudinal data to account for changes over time could enhance the robustness and accuracy of the predictive models.

## Conclusions

We implemented ML algorithms to accurately predict the risks of CVE and all-cause mortality in MHD patients. Overall, the ML models provided a more reliable prognostic assessment than traditional Cox regression models.

## Data Availability

The original contributions presented in the study are included in the article/Supplementary material, further inquiries can be directed to the corresponding author.

## References

[1] Jankowski J Floege J Fliser D Böhm M Marx N. cardiovascular disease in chronic kidney disease: pathophysiological insights and therapeutic options. Circulation. (2021) 143:1157–72. 10.1161/CIRCULATIONAHA.120.050686 33720773 PMC7969169

[2] Zhang Z Wang Y. Management of cardiovascular diseases in chronic hemodialysis patients. Rev Cardiovasc Med. (2023) 24:185. 10.31083/j.rcm2407185 39077004 PMC11266462

[3] Chinnadurai R Wu HHL Abuomar J Rengarajan S New DI Green D. Antihypertensive Prescribing patterns in non-dialysis dependent chronic kidney disease: findings from the salford kidney study. World J Nephrol. (2023) 12:168–81. 10.5527/wjn.v12.i5.168 38230298 PMC10789086

[4] Lai AC Bienstock SW Sharma R Skorecki K Beerkens F Samtani R. A personalized approach to chronic kidney disease and cardiovascular disease: jacc review topic of the week. J Am Coll Cardiol. (2021) 77:1470–9. 10.1016/j.jacc.2021.01.028 33736830

[5] Krist AH Davidson KW Mangione CM Barry MJ Cabana M Caughey AB. Behavioral counseling interventions to promote a healthy diet and physical activity for cardiovascular disease prevention in adults without cardiovascular disease risk factors us preventive services task force recommendation statement. JAMA. (2020) 324:2069–75. 10.1001/jama.2020.21749 33231670

[6] Chen R Zhang H Tang B Luo Y Yang Y Zhong X. Macrophages in cardiovascular diseases: molecular mechanisms and therapeutic targets. Signal Transd Targeted Ther. (2024) 9:130. 10.1038/s41392-024-01840-1 38816371 PMC11139930

[7] Matsushita K Ballew SH Wang AY Kalyesubula R Schaeffner E Agarwal R. Epidemiology and risk of cardiovascular disease in populations with chronic kidney disease. Nat Rev Nephrol. (2022) 18:696–707. 10.1038/s41581-022-00616-6 36104509

[8] Zoccali C Mark PB Sarafidis P Agarwal R Adamczak M Bueno de. Oliveira R, et al. Diagnosis of cardiovascular disease in patients with chronic kidney disease. Nat Rev Nephrol. (2023) 19:733–46. 10.1038/s41581-023-00747-4 37612381

[9] Marx-Schütt K Cherney DZI Jankowski J Matsushita K Nardone M Marx N. Cardiovascular disease in chronic kidney disease. Eur Heart J. (2025) 46:2148–60. 10.1093/eurheartj/ehaf167 40196891 PMC12167664

[10] Buckley LF Schmidt IM Verma A Palsson R Adam D Shah AM. Associations between kidney histopathologic lesions and incident cardiovascular disease in adults with chronic kidney disease. JAMA Cardiol. (2023) 8:357–65. 10.1001/jamacardio.2023.0056 36884237 PMC9996453

[11] Burnier M Damianaki A. Hypertension as cardiovascular risk factor in chronic kidney disease. Circ Res. (2023) 132:1050–63. 10.1161/CIRCRESAHA.122.321762 37053276

[12] Deodhare KG Pathak N. Hypertension and associated complications in pregnant women with chronic kidney disease. World J Nephrol. (2024) 13:100680. 10.5527/wjn.v13.i4.100680 39723353 PMC11572658

[13] Mohd Faizal AS Thevarajah TM Khor SM Chang SW. A review of risk prediction models in cardiovascular disease: conventional approach vs. artificial intelligent approach. Comput Meth Prog Biol. (2021) 207:106190. 10.1016/j.cmpb.2021.106190 34077865

[14] Trotsyuk AA Waeiss Q Bhatia RT Aponte BJ Heffernan IML Madgavkar D. Toward a framework for risk mitigation of potential misuse of artificial intelligence in biomedical research. Nat Mach Intell. (2024) 6:1435–42. 10.1038/s42256-024-00926-3 40994707 PMC12456743

[15] Hu JR Power JR Zannad F Lam CSP. Artificial intelligence and digital tools for design and execution of cardiovascular clinical trials. Eur Heart J. (2025) 46:814–26. 10.1093/eurheartj/ehae794 39626166

[16] van Breugel B Liu TNS Oglic D van der Schaar M. Synthetic data in biomedicine via generative artificial intelligence. Nat Rev Bioeng. (2024) 2:991–1004. 10.1038/s44222-024-00245-7

[17] Topol EJ. High-Performance medicine: the convergence of human and artificial intelligence. Nat Med. (2019) 25:44–56. 10.1038/s41591-018-0300-7 30617339

[18] Gao Q Yang L Lu M Jin R Ye H Ma T. The artificial intelligence and machine learning in lung cancer immunotherapy. J Hematol Oncol. (2023) 16:55. 10.1186/s13045-023-01456-y 37226190 PMC10207827

[19] Haug CJ Drazen JM. Artificial intelligence and machine learning in clinical medicine, 2023. N Engl J Med. (2023) 388:1201–8. 10.1056/NEJMra2302038 36988595

[20] Gupta R Kumari S Senapati A Ambasta RK Kumar P. New era of artificial intelligence and machine learning-based detection, diagnosis, and therapeutics in Parkinson's disease. Ageing Res Rev. (2023) 90:102013. 10.1016/j.arr.2023.102013 37429545

[21] Van den Eynde J Lachmann M Laugwitz KL Manlhiot C Kutty S. Successfully implemented artificial intelligence and machine learning applications in cardiology: state-of-the-art review. Trends Cardiovasc Med. (2023) 33:265–71. 10.1016/j.tcm.2022.01.010 35101642

[22] Rathore AS Nikita S Thakur G Mishra S. Artificial intelligence and machine learning applications in biopharmaceutical manufacturing. Trends Biotechnol. (2023) 41:497–510. 10.1016/j.tibtech.2022.08.007 36117026

[23] Bhat M Rabindranath M Chara BS Simonetto DA. Artificial intelligence, machine learning, and deep learning in liver transplantation. J Hepatol. (2023) 78:1216–33. 10.1016/j.jhep.2023.01.006 37208107

[24] Benjamin EJ Muntner P Alonso A Bittencourt MS Callaway CW Carson AP. Heart disease and stroke statistics-2019 update: a report from the American Heart Association. Circulation. (2019) 139:e56–528. 10.1161/CIR.0000000000000659 30700139

[25] Flores AM Demsas F Leeper NJ Ross EG. Leveraging machine learning and artificial intelligence to improve peripheral artery disease detection, treatment, and outcomes. Circ Res. (2021) 128:1833–50. 10.1161/CIRCRESAHA.121.318224 34110911 PMC8285054

[26] Jones OT Matin RN van der Schaar M Prathivadi Bhayankaram K Ranmuthu CKI Islam MS. Artificial intelligence and machine learning algorithms for early detection of skin cancer in community and primary care settings: a systematic review. Lancet Digit Health. (2022) 4:e466–76. 10.1016/S2589-7500(22)00023-1 35623799

[27] McNair D. Artificial intelligence and machine learning for lead-to-candidate decision-making and beyond. Annu Rev Pharmacol Toxicol. (2023) 63:77–97. 10.1146/annurev-pharmtox-051921-023255 35679624

[28] Wang Y Miao X Xiao G Huang C Sun J Wang Y. clinical prediction of heart failure in hemodialysis patients: based on the extreme gradient boosting method. Front Genet. (2022) 13:889378. 10.3389/fgene.2022.889378 35559036 PMC9086166

[29] Xu L Cao F Wang L Liu W Gao M Zhang L. Machine learning model and nomogram to predict the risk of heart failure hospitalization in peritoneal dialysis patients. Ren Fail. (2024) 46:2324071. 10.1080/0886022X.2024.2324071 38494197 PMC10946267

[30] Akbilgic O Obi Y Potukuchi PK Karabayir I Nguyen DV Soohoo M. Machine learning to identify dialysis patients at high death risk. Kidney Int Rep. (2019) 4:1219–29. 10.1016/j.ekir.2019.06.009 31517141 PMC6732773

[31] Sheng K Zhang P Yao X Li J He Y Chen J. Prognostic machine learning models for first-year mortality in incident hemodialysis patients: development and validation study. JMIR Med Inform. (2020) 8:e20578. 10.2196/20578 33118948 PMC7661257

[32] Bai P Zhou Y Liu Y Li G Li Z Wang T. Risk factors of cerebral infarction and myocardial infarction after carotid endarterectomy analyzed by machine learning. Comput Math Method. (2020) 2020:6217392. 10.1155/2020/6217392 33273961 PMC7683166

[33] Zhao BR Hu XR Wang WD Zhou Y. Cardiorenal syndrome: clinical diagnosis, molecular mechanisms and therapeutic strategies. Acta Pharmacol Sin. (2025) 46:1539–55. 10.1038/s41401-025-01476-z 39910210 PMC12098865

[34] Stirnadel-Farrant HA Karaboyas A Cizman B Bieber BA Kler L Jones D. Cardiovascular event rates among hemodialysis patients across geographical regions-a snapshot from the dialysis outcomes and practice patterns study (dopps). Kidney Int Rep. (2019) 4:864–72. 10.1016/j.ekir.2019.03.016 31194073 PMC6551512

[35] Barad A Clark AG Pressman EK O'Brien KO. Associations between genetically predicted iron status and cardiovascular disease risk: a mendelian randomization study. J Am Heart Assoc. (2024) 13:e034991. 10.1161/JAHA.124.034991 38818967 PMC11255641

[36] Li D Wang A Li Y Ruan Z Zhao H Li J. et al. Nonlinear relationship of red blood cell indices (Mch, Mchc, and Mcv) with all-cause and cardiovascular mortality: a cohort study in US adults. PLoS ONE. (2024) 19:e0307609. 10.1371/journal.pone.0307609 39093828 PMC11296621

[37] Sakaguchi Y Fujii N Shoji T Hayashi T Rakugi H Isaka Y. Hypomagnesemia Is a significant predictor of cardiovascular and non-cardiovascular mortality in patients undergoing hemodialysis. Kidney Int. (2014) 85:174–81. 10.1038/ki.2013.327 23986148

[38] Soohoo M Streja E Hsiung JT Kovesdy CP Kalantar-Zadeh K Arah OA. Cohort study and bias analysis of the obesity paradox across stages of chronic kidney disease. J Renal Nutr. (2022) 32:529–36. 10.1053/j.jrn.2021.10.007 34861399 PMC10032545

[39] Zhao X Wu Y Lee DL Cui W. Iforest: interpreting random forests via visual analytics. IEEE Vis Comput Gr. (2018) 25:407–16. 10.1109/TVCG.2018.2864475 30188822

[40] Chen M Zeng Y Liu M Li Z Wu J Tian X. Interpretable machine learning models for the prediction of all-cause mortality and time to death in hemodialysis patients. Ther Apher Dial. (2025) 29:220–32. 10.1111/1744-9987.14212 39327762 PMC11879476

[41] Sumida K Yamagata K Iseki K Tsubakihara Y. Different impact of hemodialysis vintage on cause-specific mortality in long-term hemodialysis patients. Nephrol Dial Transpl. (2016) 31:298–305. 10.1093/ndt/gfv402 26666499

[42] Cheung AK Greene T Leypoldt JK Yan G Allon M Delmez J. Association between serum 2-microglobulin level and infectious mortality in hemodialysis patients. Clin J Am Soc Nephrol. (2008) 3:69–77. 10.2215/CJN.02340607 18057309 PMC2390979

[43] Gotta V Tancev G Marsenic O Vogt JE Pfister M. identifying key predictors of mortality in young patients on chronic haemodialysis-a machine learning approach. Nephrol Dial Transpl. (2021) 36:519–28. 10.1093/ndt/gfaa128 32510143

[44] Shah AD Denaxas S Nicholas O Hingorani AD Hemingway H. Low eosinophil and low lymphocyte counts and the incidence of 12 cardiovascular diseases: a caliber cohort study. Open Heart. (2016) 3:e000477. 10.1136/openhrt-2016-000477 27621833 PMC5013342

[45] Xiong L Chen QQ Cheng Y Lan YS Yang JB Wen XQ. The relationship between coronary artery calcification and bone metabolic markers in maintenance hemodialysis patients. BMC Nephrol. (2023) 24:238. 10.1186/s12882-023-03286-z 37582785 PMC10428586

[46] Linardatos P Papastefanopoulos V Kotsiantis S. Explainable AI: a review of machine learning interpretability methods. Entropy (Basel). (2020) 23:1–45. 10.3390/e23010018 33375658 PMC7824368

[47] Raikou VD. Renoprotective strategies. World J Nephrol. (2024) 13:89637. 10.5527/wjn.v13.i1.89637 38596266 PMC11000037

[48] Miao H Wang YN Su W Zou L Zhuang SG Yu XY. Sirtuin 6 protects against podocyte injury by blocking the renin-angiotensin system by inhibiting the Wnt1/?-catenin pathway. Acta Pharmacol Sin. (2024) 45:137–49. 10.1038/s41401-023-01148-w 37640899 PMC10770168

[49] Liu B Shalamu A Pei Z Liu L Wei Z Qu Y. A novel mouse model of heart failure with preserved ejection fraction after chronic kidney disease induced by retinol through Jak/Stat pathway. Int J Biol Sci. (2023) 19:3661–77. 10.7150/ijbs.83432 37564202 PMC10411473

[50] Liu T Li Q Jin Q Yang L Mao H Qu P. Targeting Hmgb1: a potential therapeutic strategy for chronic kidney disease. Int J Biol Sci. (2023) 19:5020–35. 10.7150/ijbs.87964 37781525 PMC10539693

[51] Liu D Chen X He W Lu M Li Q Zhang S. Update on the pathogenesis, diagnosis, and treatment of diabetic tubulopathy. Integr Med Nephrol Androl. (2024) 11:e23–00029. 10.1097/IMNA-D-23-00029

[52] Wang YN Zhang ZH Liu HJ Guo ZY Zou L Zhang YM. Integrative phosphatidylcholine metabolism through phospholipase a(2) in rats with chronic kidney disease. Acta Pharmacol Sin. (2023) 44:393–405. 10.1038/s41401-022-00947-x 35922553 PMC9889763

[53] Wang YN Miao H Yu XY Guo Y Su W Liu F. Oxidative stress and inflammation are mediated via aryl hydrocarbon receptor signalling in idiopathic membranous nephropathy. Free Radic Biol Med. (2023) 207:89–106. 10.1016/j.freeradbiomed.2023.07.014 37451370

[54] Luo M Cai J Luo S Hong X Xu L Lin H. Causal effects of gut microbiota on the risk of chronic kidney disease: a mendelian randomization study. Front Cell Infect Microbiol. (2023) 13:1142140. 10.3389/fcimb.2023.1142140 37065213 PMC10102584

[55] Zhang J Zhu P Li S Gao Y Xing Y. From heart failure and kidney dysfunction to cardiorenal syndrome: Tmao may be a bridge. Front Pharmacol. (2023) 14:1291922. 10.3389/fphar.2023.1291922 38074146 PMC10703173

[56] Tao P Huo J Chen L. Bibliometric analysis of the relationship between gut microbiota and chronic kidney disease from 2001–2022. Integr Med Nephrol Androl. (2024) 11:e00017. 10.1097/IMNA-D-23-00017

[57] Li XJ Shan QY Wu X Miao H Zhao YY. Gut microbiota regulates oxidative stress and inflammation: a double-edged sword in renal fibrosis. Cell Mol Life Sci. (2024) 81:480. 10.1007/s00018-024-05532-5 39636415 PMC11621299

[58] Rong J Zhang Z Peng X Li P Zhao T Zhong Y. Mechanisms of hepatic and renal injury in lipid metabolism disorders in metabolic syndrome. Int J Biol Sci. (2024) 20:4783–98. 10.7150/ijbs.100394 39309427 PMC11414397

