# Supplementary material for: Risk prediction for cardiovascular events and all-cause mortality in maintenance hemodialysis patients
Source: Front Med (Lausanne). 2025 Oct 27;12:1660154. doi: 10.3389/fmed.2025.1660154 (PMC12597953; doi:10.3389/fmed.2025.1660154)
Supplement: Supplementary file 1 [file Table_1.docx]

Supplementary Material

**Supplementary Tables**

**Supplementary Table 1 Specific variables of data set.**

|  | Variables | Overall (n=275) |
| --- | --- | --- |
| Demographic characteristics | Gender | Male: 171 (62.2%) |
|  |  | Female: 104 (37.8%) |
|  | Age (years old) | 56.00 (48.00, 67.00) |
|  | Dialysis Vintage (months) | 64.00 (41.00, 92.00) |
|  | Pre-dialysis Systolic Blood Pressure (mmHg) | 139.28 (18.95) |
|  | Pre-dialysis Diastolic Blood Pressure (mmHg) | 80.80 (13.74) |
|  | Pre-dialysis Pulse (per minute) | 80.00 (70.00, 87.00) |
|  | Body Mass Index (Kg/m^2^) | 22.78 (20.77, 24.80) |
|  | Smoking | 103 (37.5%) |
|  | Driking | 80 (29.1%) |
| Comorbidities | Hypertension | 171 (62.2%) |
|  | Diabetes | 96 (34.9%) |
|  | Hyperlipidaemia | 166 (60.4%) |
|  | Hyperuricaemia | 117 (42.5%) |
|  | Osteoporosis | 99 (36.0%) |
|  | History of Cardiovascular Disease | 74 (26.9%) |
| Medication history | Calcium Carbonate | 196 (71.3%) |
|  | Lanthanum Carbonate | 149 (54.2%) |
|  | Cinacalcet | 115 (41.8%) |
|  | Sevelamer | 226 (82.2%) |
|  | Osteotriol | 198 (72.0%) |
|  | Paricalcitol | 178 (64.7%) |
|  | Rosadustat | 81 (29.5%) |
|  | Febuxostat | 107 (38.9%) |
|  | Lipid-lowering Drugs | 176 (64.0%) |
| Laboratory values | White Blood Cell Count (×10⁹/L) | 6.09 (5.16, 7.64) |
|  | Neutrophil Count (×10⁹/L) | 4.30 (3.45, 5.57) |
|  | Lymphocyte Count (×10⁹/L) | 1.04 (0.86, 1.31) |
|  | Monocyte Count (×10⁹/L) | 0.37 (0.29, 0.46) |
|  | Neutrophil Ratio | 71.80 (66.50, 76.30) |
|  | Red Blood Cell Count (×10¹²/L) | 3.52 (0.65) |
|  | Hemoglobin (g/L) | 107.00 (94.00, 118.00) |
|  | Mean Corpuscular Volume (fl) | 93.80 (89.95, 97.65) |
|  | Mean Corpuscular Hemoglobin (pg) | 30.10 (28.80, 31.70) |
|  | Mean Corpuscular Hemoglobin Concentration (g/L) | 322.00 (314.50, 327.50) |
|  | Red Cell Distribution Width-Standard Deviation (fl) | 46.50 (44.10, 49.25) |
|  | Red Cell Distribution Width-Coefficient of Variation (%) | 14.00 (13.30, 14.90) |
|  | Platelet Count (×10⁹/L) | 166.00 (131.50, 214.00) |
|  | Platelet Crit | 0.16 (0.13, 0.20) |
|  | Platelet Distribution Width | 16.10 (15.80, 16.40) |
|  | High-sensitivity C-Reactive Protein (mg/L) | 1.44 (0.53, 4.05) |
|  | Serum Amyloid A (mg/L) | 6.02 (3.38, 12.01) |
|  | Alanine Aminotransferase (U/L) | 11.80 (8.30, 18.30) |
|  | Aspartate Aminotransferase (U/L) | 13.70 (10.10, 18.20) |
|  | Total Protein (g/L) | 65.80 (62.50, 69.60) |
|  | Albumin (g/L) | 39.10 (36.70, 41.00) |
|  | Total Bilirubin (umol/L) | 5.60 (4.40, 7.00) |
|  | Direct Bilirubin (umol/L) | 2.10 (1.60, 2.70) |
|  | Indirect Bilirubin (umol/L) | 3.50 (2.50, 4.50) |
|  | Total Bile Acids (umol/L) | 4.90 (3.25, 7.55) |
|  | Lactate Dehydrogenase (U/L) | 190.30 (164.90, 225.15) |
|  | Gamma-Glutamyl Transferase (U/L) | 20.30 (13.50, 35.05) |
|  | Alkaline Phosphatase (U/L) | 88.00 (67.65, 114.45) |
|  | Prealbumin (mg/L) | 295.50 (256.75, 347.85) |
|  | Blood Urea Nitrogen (mmol/L) | 23.43 (18.04, 28.43) |
|  | Uric Acid (umol/L) | 464.90 (405.10, 524.60) |
|  | Serum Creatinine (umol/L) | 816.30 (667.25, 970.50) |
|  | Total Cholesterol (mmol/L) | 3.59 (3.00, 4.34) |
|  | Triglycerides (mmol/L) | 1.52 (1.11, 2.26) |
|  | High-Density Lipoprotein Cholesterol (mmol/L) | 1.03 (0.87, 1.25) |
|  | Low-Density Lipoprotein Cholesterol (mmol/L) | 1.90 (1.48, 2.55) |
|  | Apolipoprotein A1 (g/L) | 1.22 (1.06, 1.37) |
|  | Apolipoprotein B (g/L) | 0.67 (0.53, 0.82) |
|  | Glucose (mmol/L) | 7.13 (5.77, 8.80) |
|  | Potassium (mmol/L) | 4.69 (4.26, 5.38) |
|  | Sodium (mmol/L) | 138.60 (135.90, 141.10) |
|  | Calcium (mmol/L) | 2.20 (2.08, 2.31) |
|  | Phosphate (mmol/L) | 1.65 (1.30, 2.08) |
|  | Calcium × Phosphate Product (mg²/dL²) | 45.91 (35.02, 56.92) |
|  | Magnesium (mmol/L) | 1.00 (0.89, 1.15) |
|  | Iron (mmol/L) | 9.40 (7.40, 11.70) |
|  | Unsaturated Iron Binding Capacity (umol/L) | 31.00 (26.10, 37.30) |
|  | Total Iron Binding Capacity (umol/L) | 40.50 (35.50, 47.40) |
|  | Reticulocyte Binding Protein (mg/L) | 99.70 (90.95, 107.35) |
|  | Estimated Glomerular Filtration Rate (ml/min) | 5.10 (4.15, 6.75) |
|  | Ferritin (ng/mL) | 272.73 (127.03, 455.14) |
|  | Calcitonin (pg/mL) | 8.14 (2.89, 18.30) |
|  | Parathyroid Hormone (pg/mL) | 386.41 (222.96, 578.96) |
|  | 25-Hydroxy Vitamin D (ng/mL) | 13.54 (10.00, 18.06) |
|  | Beta-CrossLaps (pg/mL) | 2096.00 (1282.00, 2970.00) |
|  | Osteocalcin (ng/mL) | 164.70 (97.94, 213.70) |
|  | Total Procollagen I Amino-terminal Propeptide (ng/mL) | 333.30 (213.90, 554.10) |
|  | Pre-dialysis Beta-2-Microglobulin (mg/L) | 30.40 (25.21, 35.97) |
|  | Post-dialysis Beta-2-Microglobulin (mg/L) | 11.34 (8.63, 14.61) |
|  | High-sensitivity Troponin T (ug/L) | 0.05 (0.03, 0.08) |
|  | Myoglobin (ug/L) | 165.60 (125.92, 227.15) |
|  | Creatine Kinase - MB (ug/L) | 1.60 (1.10, 2.25) |
|  | N-Terminal Pro B-Type Natriuretic Peptide (ng/L) | 6842.00 (2485.00, 18039.50) |

Measures that conform to normal distribution are expressed as mean ± standard deviation, and those that do not conform to normal distribution are expressed as median (interquartile spacing). Count data are expressed as frequency (percentage %).

**Supplementary Table 2 Comparison of baseline date in patients between cardiovascular events group and non-** **cardiovascular events group.**

| Variables | CVE group | non-CVE group | *P* value |
| --- | --- | --- | --- |
| Gender | Male:80 (67.2%) | Male:91 (58.3%) | 0.167 |
|  | Famale:39 (32.8%) | Famale:65 (41.7%) |  |
| Age (years old) | 62.00(53.00, 69.05) | 54.00(44.00, 64.00) | <0.001 |
| Dialysis Vintage (months) | 62.00(38.00, 89.00) | 67.00(42.75, 96.25) | 0.086 |
| Pre-dialysis Systolic Blood Pressure (mmHg) | 141.47±19.63 | 137.61±18.31 | 0.094 |
| Pre-dialysis Diastolic Blood Pressure (mmHg) | 80.62±15.26 | 80.94±12.50 | 0.851 |
| Pre-dialysis Pulse (per minute) | 80.00 (70.00, 88.00) | 80.00 (69.75, 86.00) | 0.652 |
| Body Mass Index (Kg/m^2^) | 23.12 (20.99, 24.85) | 22.40 (20.48, 24.75) | 0.166 |
| Smoking | 51 (42.9%) | 52 (33.3%) | 0.136 |
| Driking | 41 (34.5%) | 39 (25.0%) | 0.115 |
| Hypertension | 87 (73.1%) | 84 (53.8%) | 0.002 |
| Diabetes | 52 (43.7%) | 44 (28.2%) | 0.011 |
| Hyperlipidaemia | 76 (63.9%) | 90 (57.7%) | 0.361 |
| Hyperuricaemia | 44 (37.0%) | 73 (46.8%) | 0.131 |
| Osteoporosis | 47 (39.5%) | 52 (33.3%) | 0.353 |
| History of Cardiovascular Disease | 51 (42.9%) | 23 (14.7%) | <0.001 |
| Calcium Carbonate | 80 (67.2%) | 116 (74.4%) | 0.246 |
| Lanthanum Carbonate | 61 (51.3%) | 88 (56.4%) | 0.467 |
| Cinacalcet | 41 (34.5%) | 74 (47.4%) | 0.041 |
| Sevelamer | 95 (79.8%) | 131 (84.0%) | 0.465 |
| Osteotriol | 87 (73.1%) | 111 (71.2%) | 0.824 |
| Paricalcitol | 70 (58.8%) | 108 (69.2%) | 0.096 |
| Rosadustat | 35 (29.4%) | 46 (29.5%) | 1.000 |
| Febuxostat | 40 (33.6%) | 67 (42.9%) | 0.148 |
| Lipid-lowering Drugs | 86 (72.3%) | 90 (57.7%) | 0.018 |
| White Blood Cell Count (×10⁹/L) | 6.40 (5.30, 7.94) | 5.89 (4.92, 7.25) | 0.032 |
| Neutrophil Count (×10⁹/L) | 4.44 (3.67, 6.12) | 4.22 (3.32, 5.28) | 0.085 |
| Lymphocyte Count (×10⁹/L) | 1.06 (0.91, 1.28) | 1.01 (0.80, 1.31) | 0.396 |
| Monocyte Count (×10⁹/L) | 0.39 (0.31, 0.49) | 0.36 (0.27, 0.44) | 0.006 |
| Neutrophil Ratio | 71.70 (67.30, 76.30) | 71.80 (66.22, 75.95) | 0.828 |
| Red Blood Cell Count (×10¹²/L) | 3.52±0.67 | 3.53±0.64 | 0.840 |
| Hemoglobin (g/L) | 103.00 (93.00, 115.00) | 110.00 (94.75, 119.00) | 0.174 |
| Mean Corpuscular Volume (fl) | 93.20 (89.35, 97.35) | 94.55 (90.50, 97.93) | 0.082 |
| Mean Corpuscular Hemoglobin (pg) | 29.80 (28.70, 31.40) | 30.40 (29.00, 31.70) | 0.074 |
| Mean Corpuscular Hemoglobin Concentration (g/L) | 322.00 (315.00, 328.50) | 322.00 (314.00, 326.25) | 0.531 |
| Red Cell Distribution Width-Standard Deviation (fl) | 47.10 (44.05, 49.70) | 46.20 (44.18, 49.00) | 0.764 |
| Red Cell Distribution Width-Coefficient of Variation (%) | 14.20 (13.50, 15.20) | 13.80 (13.17, 14.70) | 0.008 |
| Platelet Count (×10⁹/L) | 169.00 (136.50, 225.00) | 161.00 (126.00, 208.00) | 0.122 |
| Platelet Crit | 0.17 (0.13, 0.20) | 0.15 (0.13, 0.19) | 0.077 |
| Platelet Distribution Width | 16.10 (15.80, 16.30) | 16.10 (15.80, 16.40) | 0.541 |
| High-sensitivity C-Reactive Protein (mg/L) | 1.85 (0.68, 5.58) | 1.07 (0.50, 3.41) | 0.015 |
| Serum Amyloid A (mg/L) | 7.90 (3.81, 14.12) | 5.11 (3.01, 9.94) | 0.030 |
| Alanine Aminotransferase (U/L) | 11.60 (8.00, 15.45) | 11.80 (8.47, 19.12) | 0.270 |
| Aspartate Aminotransferase (U/L) | 12.90 (9.70, 16.40) | 14.20 (10.47, 18.80) | 0.091 |
| Total Protein (g/L) | 65.80 (62.60, 69.65) | 65.70 (62.22, 69.38) | 0.593 |
| Albumin (g/L) | 38.80 (36.60, 40.90) | 39.20 (36.88, 41.02) | 0.775 |
| Total Bilirubin (umol/L) | 5.60 (4.40, 6.85) | 5.60 (4.30, 7.05) | 0.944 |
| Direct Bilirubin (umol/L) | 2.00 (1.50, 2.70) | 2.15 (1.60, 2.80) | 0.426 |
| Indirect Bilirubin (umol/L) | 3.50 (2.70, 4.50) | 3.40 (2.50, 4.43) | 0.490 |
| Total Bile Acids (umol/L) | 5.30 (3.45, 7.55) | 4.85 (3.10, 7.40) | 0.545 |
| Lactate Dehydrogenase (U/L) | 192.70 (166.40, 224.25) | 189.50 (164.48, 224.92) | 0.915 |
| Gamma-Glutamyl Transferase (U/L) | 21.30 (14.20, 36.40) | 19.90 (12.80, 30.92) | 0.170 |
| Alkaline Phosphatase (U/L) | 89.40 (68.65, 115.40) | 87.40 (67.57, 113.82) | 0.956 |
| Prealbumin (mg/L) | 288.70 (245.70, 340.05) | 302.15 (259.68, 353.45) | 0.172 |
| Blood Urea Nitrogen (mmol/L) | 23.04 (18.16, 26.72) | 23.83 (17.93, 29.31) | 0.540 |
| Uric Acid (umol/L) | 466.00 (405.55, 537.15) | 460.65 (402.88, 517.90) | 0.556 |
| Serum Creatinine (umol/L) | 800.00 (640.95, 966.15) | 823.10 (696.45, 975.30) | 0.253 |
| Total Cholesterol (mmol/L) | 3.63 (3.11, 4.36) | 3.58 (2.96, 4.32) | 0.449 |
| Triglycerides (mmol/L) | 1.56 (1.14, 2.29) | 1.51 (1.08, 2.12) | 0.324 |
| High-Density Lipoprotein Cholesterol (mmol/L) | 1.01 (0.88, 1.29) | 1.06 (0.86, 1.24) | 0.965 |
| Low-Density Lipoprotein Cholesterol (mmol/L) | 1.89 (1.48, 2.52) | 1.90 (1.53, 2.55) | 0.786 |
| Apolipoprotein A1 (g/L) | 1.22 (1.09, 1.38) | 1.22 (1.05, 1.37) | 0.703 |
| Apolipoprotein B (g/L) | 0.67 (0.54, 0.83) | 0.66 (0.53, 0.80) | 0.390 |
| Glucose (mmol/L) | 7.21 (5.79, 9.38) | 7.04 (5.77, 8.74) | 0.324 |
| Potassium (mmol/L) | 4.64 (4.19, 5.30) | 4.76 (4.34, 5.43) | 0.123 |
| Sodium (mmol/L) | 138.60 (135.90, 140.55) | 138.85 (135.88, 141.40) | 0.423 |
| Calcium (mmol/L) | 2.20 (2.08, 2.35) | 2.20 (2.07, 2.30) | 0.949 |
| Phosphate (mmol/L) | 1.65 (1.33, 2.09) | 1.63 (1.30, 2.06) | 0.935 |
| Calcium × Phosphate Product (mg²/dL²) | 46.36 (36.54, 57.22) | 44.79 (34.11, 56.47) | 0.497 |
| Magnesium (mmol/L) | 0.98 (0.88, 1.13) | 1.01 (0.90, 1.16) | 0.216 |
| Iron (mmol/L) | 9.30 (7.15, 11.05) | 9.45 (7.57, 11.90) | 0.303 |
| Unsaturated Iron Binding Capacity (umol/L) | 30.20 (26.15, 36.35) | 31.80 (26.05, 38.28) | 0.443 |
| Total Iron Binding Capacity (umol/L) | 39.80 (34.95, 45.95) | 40.80 (35.98, 48.40) | 0.122 |
| Reticulocyte Binding Protein (mg/L) | 100.00 (89.80, 106.15) | 99.55 (91.18, 108.70) | 0.418 |
| Estimated Glomerular Filtration Rate (ml/min) | 5.00 (4.15, 7.10) | 5.10 (4.18, 6.53) | 0.627 |
| Ferritin (ng/mL) | 277.71 (129.12, 515.12) | 258.93 (127.09, 422.27) | 0.282 |
| Calcitonin (pg/mL) | 8.62 (3.67, 18.28) | 7.54 (2.32, 18.28) | 0.282 |
| Parathyroid Hormone (pg/mL) | 358.09 (221.14, 549.36) | 400.29 (229.60, 617.70) | 0.254 |
| 25-Hydroxy Vitamin D (ng/mL) | 11.85 (9.28, 18.24) | 13.91 (10.70, 18.00) | 0.164 |
| Beta-CrossLaps (pg/mL) | 1835.00 (1148.00, 2795.50) | 2216.00 (1423.25, 3084.50) | 0.011 |
| Osteocalcin (ng/mL) | 155.70 (80.20, 220.60) | 175.45 (106.55, 211.65) | 0.445 |
| Total Procollagen I Amino-terminal Propeptide (ng/mL) | 312.60 (187.70, 570.20) | 367.20 (228.45, 529.68) | 0.120 |
| Pre-dialysis Beta-2-Microglobulin (mg/L) | 30.16 (25.06, 34.40) | 30.68 (25.22, 36.74) | 0.364 |
| Post-dialysis Beta-2-Microglobulin (mg/L) | 11.54 (8.91, 15.47) | 11.27 (8.01, 14.38) | 0.223 |
| High-sensitivity Troponin T (ug/L) | 0.06 (0.04, 0.08) | 0.04 (0.03, 0.07) | <0.001 |
| Myoglobin (ug/L) | 166.10 (124.60, 233.80) | 165.20 (128.50, 214.40) | 0.934 |
| Creatine Kinase - MB (ug/L) | 1.88 (1.27, 2.60) | 1.41 (1.02, 2.13) | 0.002 |
| N-Terminal Pro B-Type Natriuretic Peptide (ng/L) | 10725.00 (3169.00, 32667.00) | 4626.00 (2341.75, 11151.75) | <0.001 |

**Supplementary Table 3 Comparison of baseline data in patients between death and survival groups.**

| Variables | Death group | Survival group | *P* value |
| --- | --- | --- | --- |
| Gender | Male:52 (69.3%) | Male:119 (59.5%) | 0.174 |
|  | Famale:23 (30.7%) | Famale:81 (40.5%) |  |
| Age (years old) | 64.00 (53.00, 70.50) | 55.00 (46.00, 65.00) | <0.001 |
| Dialysis Vintage (months) | 51.00 (25.00, 70.00) | 67.00 (47.75, 105.50) | <0.001 |
| Pre-dialysis Systolic Blood Pressure (mmHg) | 140.59±20.14 | 138.79±18.52 | 0.485 |
| Pre-dialysis Diastolic Blood Pressure (mmHg) | 77.19±13.73 | 82.16±13.53 | 0.007 |
| Pre-dialysis Pulse (per minute) | 80.00 (72.00, 87.00) | 80.00 (69.75, 87.00) | 0.424 |
| Body Mass Index (Kg/m^2^) | 22.77 (20.35, 24.49) | 22.80 (20.90, 24.90) | 0.923 |
| Smoking | 38 (50.7%) | 65 (32.5%) | 0.008 |
| Driking | 32 (42.7%) | 48 (24.0%) | 0.004 |
| Hypertension | 50 (66.7%) | 121 (60.5%) | 0.424 |
| Diabetes | 33 (44.0%) | 63 (31.5%) | 0.073 |
| Hyperlipidaemia | 42 (56.0%) | 124 (62.0%) | 0.443 |
| Hyperuricaemia | 24 (32.0%) | 93 (46.5%) | 0.042 |
| Osteoporosis | 29 (38.7%) | 70 (35.0%) | 0.672 |
| History of Cardiovascular Disease | 29 (38.7%) | 45 (22.5%) | 0.011 |
| Calcium Carbonate | 48 (64.0%) | 148 (74.0%) | 0.138 |
| Lanthanum Carbonate | 32 (42.7%) | 117 (58.5%) | 0.027 |
| Cinacalcet | 17 (22.7%) | 98 (49.0%) | <0.001 |
| Sevelamer | 57 (76.0%) | 169 (84.5%) | 0.143 |
| Osteotriol | 52 (69.3%) | 146 (73.0%) | 0.651 |
| Paricalcitol | 27 (36.0%) | 151 (75.5%) | <0.001 |
| Rosadustat | 13 (17.3%) | 68 (34.0%) | 0.011 |
| Febuxostat | 16 (21.3%) | 91 (45.5%) | <0.001 |
| Lipid-lowering Drugs | 50 (66.7%) | 126 (63.0%) | 0.672 |
| White Blood Cell Count (×10⁹/L) | 6.50 (5.38, 8.14) | 5.92 (5.10, 7.43) | 0.089 |
| Neutrophil Count (×10⁹/L) | 4.64 (3.62, 6.22) | 4.22 (3.44, 5.44) | 0.133 |
| Lymphocyte Count (×10⁹/L) | 1.06 (0.83, 1.20) | 1.04 (0.87, 1.32) | 0.659 |
| Monocyte Count (×10⁹/L) | 0.40 (0.31, 0.52) | 0.36 (0.28, 0.44) | 0.012 |
| Neutrophil Ratio | 71.30 (66.85, 76.35) | 71.85 (66.28, 75.88) | 0.972 |
| Red Blood Cell Count (×10¹²/L) | 3.47±0.69 | 3.54±0.64 | 0.410 |
| Hemoglobin (g/L) | 103.00 (91.00, 115.50) | 108.50 (94.75, 118.25) | 0.196 |
| Mean Corpuscular Volume (fl) | 92.90 (89.60, 97.95) | 94.20 (90.22, 97.60) | 0.630 |
| Mean Corpuscular Hemoglobin (pg) | 30.10 (28.80, 31.70) | 30.10 (28.90, 31.63) | 0.699 |
| Mean Corpuscular Hemoglobin Concentration (g/L) | 321.00 (313.50, 327.00) | 322.00 (315.00, 328.00) | 0.795 |
| Red Cell Distribution Width-Standard Deviation (fl) | 46.70 (44.20, 49.25) | 46.50 (44.08, 49.25) | 0.696 |
| Red Cell Distribution Width-Coefficient of Variation (%) | 14.10 (13.45, 14.90) | 13.90 (13.28, 15.00) | 0.837 |
| Platelet Count (×10⁹/L) | 166.00 (123.50, 204.00) | 167.00 (135.00, 215.75) | 0.264 |
| Platelet Crit | 0.16 (0.12, 0.18) | 0.16 (0.13, 0.20) | 0.170 |
| Platelet Distribution Width | 16.10 (15.70, 16.40) | 16.10 (15.80, 16.40) | 0.850 |
| High-sensitivity C-Reactive Protein (mg/L) | 2.34 (0.94, 5.41) | 1.17 (0.50, 3.60) | 0.012 |
| Serum Amyloid A (mg/L) | 8.27 (4.00, 14.45) | 5.28 (3.08, 10.61) | 0.031 |
| Alanine Aminotransferase (U/L) | 11.60 (8.65, 16.50) | 11.85 (8.10, 18.50) | 0.980 |
| Aspartate Aminotransferase (U/L) | 13.40 (9.55, 18.50) | 13.75 (10.20, 17.95) | 0.951 |
| Total Protein (g/L) | 65.80 (63.15, 69.35) | 65.80 (62.00, 69.60) | 0.756 |
| Albumin (g/L) | 38.10 (35.65, 39.60) | 39.40 (37.20, 41.20) | 0.002 |
| Total Bilirubin (umol/L) | 5.80 (4.25, 7.50) | 5.60 (4.40, 6.73) | 0.830 |
| Direct Bilirubin (umol/L) | 2.30 (1.60, 3.15) | 2.10 (1.60, 2.60) | 0.210 |
| Indirect Bilirubin (umol/L) | 3.20 (2.35, 4.55) | 3.50 (2.60, 4.43) | 0.284 |
| Total Bile Acids (umol/L) | 4.60 (3.40, 7.35) | 5.10 (3.10, 7.60) | 0.915 |
| Lactate Dehydrogenase (U/L) | 188.60 (168.00, 226.65) | 190.60 (163.10, 220.27) | 0.544 |
| Gamma-Glutamyl Transferase (U/L) | 26.50 (14.85, 48.30) | 19.50 (13.10, 29.75) | 0.003 |
| Alkaline Phosphatase (U/L) | 94.40 (76.05, 123.80) | 85.30 (64.50, 113.35) | 0.063 |
| Prealbumin (mg/L) | 277.80 (230.20, 327.15) | 302.60 (264.38, 352.22) | 0.005 |
| Blood Urea Nitrogen (mmol/L) | 22.81 (17.70, 26.46) | 23.58 (18.35, 29.46) | 0.133 |
| Uric Acid (umol/L) | 452.90 (388.95, 507.60) | 465.85 (408.90, 530.88) | 0.131 |
| Serum Creatinine (umol/L) | 736.20 (604.35, 859.90) | 842.90 (696.50, 1001.25) | <0.001 |
| Total Cholesterol (mmol/L) | 3.50 (2.94, 4.25) | 3.63 (3.11, 4.38) | 0.216 |
| Triglycerides (mmol/L) | 1.38 (1.00, 2.06) | 1.58 (1.14, 2.43) | 0.092 |
| High-Density Lipoprotein Cholesterol (mmol/L) | 1.01 (0.86, 1.34) | 1.04 (0.88, 1.24) | 0.601 |
| Low-Density Lipoprotein Cholesterol (mmol/L) | 1.72 (1.42, 2.60) | 1.98 (1.54, 2.54) | 0.204 |
| Apolipoprotein A1 (g/L) | 1.20 (1.06, 1.38) | 1.23 (1.07, 1.37) | 0.714 |
| Apolipoprotein B (g/L) | 0.60 (0.50, 0.78) | 0.68 (0.54, 0.82) | 0.071 |
| Glucose (mmol/L) | 7.67 (6.19, 9.51) | 7.06 (5.71, 8.75) | 0.093 |
| Potassium (mmol/L) | 4.57 (4.12, 5.32) | 4.74 (4.32, 5.39) | 0.105 |
| Sodium (mmol/L) | 137.50 (135.75, 140.90) | 138.80 (136.17, 141.22) | 0.420 |
| Calcium (mmol/L) | 2.19 (2.06, 2.30) | 2.21 (2.08, 2.31) | 0.415 |
| Phosphate (mmol/L) | 1.52 (1.27, 2.02) | 1.71 (1.31, 2.09) | 0.151 |
| Calcium × Phosphate Product (mg²/dL²) | 42.83 (33.88, 56.30) | 47.29 (36.42, 56.83) | 0.222 |
| Magnesium (mmol/L) | 0.98 (0.86, 1.12) | 1.01 (0.90, 1.16) | 0.146 |
| Iron (mmol/L) | 9.50 (6.95, 11.10) | 9.35 (7.47, 11.80) | 0.613 |
| Unsaturated Iron Binding Capacity (umol/L) | 28.00 (25.10, 34.80) | 32.40 (27.08, 37.52) | 0.005 |
| Total Iron Binding Capacity (umol/L) | 38.40 (33.75, 42.90) | 41.20 (36.00, 48.23) | 0.004 |
| Reticulocyte Binding Protein (mg/L) | 95.90 (89.00, 104.15) | 100.90 (91.83, 108.73) | 0.006 |
| Estimated Glomerular Filtration Rate (ml/min) | 5.50 (4.60, 7.30) | 4.90 (4.00, 6.43) | 0.008 |
| Ferritin (ng/mL) | 312.65 (170.44, 507.03) | 241.00 (106.10, 424.63) | 0.032 |
| Calcitonin (pg/mL) | 11.11 (4.38, 26.50) | 7.34 (2.34, 15.62) | 0.019 |
| Parathyroid Hormone (pg/mL) | 374.34 (227.03, 560.26) | 388.64 (221.61, 593.53) | 0.796 |
| 25-Hydroxy Vitamin D (ng/mL) | 12.45 (9.05, 18.78) | 13.61 (10.51, 17.92) | 0.592 |
| Beta-CrossLaps (pg/mL) | 1829.00 (1150.00, 2799.00) | 2192.50 (1339.25, 2995.25) | 0.035 |
| Osteocalcin (ng/mL) | 160.10 (80.21, 209.30) | 168.50 (104.78, 214.50) | 0.414 |
| Total Procollagen I Amino-terminal Propeptide (ng/mL) | 290.30 (178.50, 565.30) | 353.35 (218.53, 542.93) | 0.115 |
| Pre-dialysis Beta-2-Microglobulin (mg/L) | 30.63 (25.71, 36.16) | 30.37 (24.88, 35.41) | 0.906 |
| Post-dialysis Beta-2-Microglobulin (mg/L) | 12.54 (9.78, 15.47) | 10.75 (8.26, 14.43) | 0.014 |
| High-sensitivity Troponin T (ug/L) | 0.07 (0.05, 0.11) | 0.04 (0.03, 0.07) | <0.001 |
| Myoglobin (ug/L) | 156.40 (124.80, 233.90) | 167.30 (128.50, 216.00) | 0.954 |
| Creatine Kinase - MB (ug/L) | 1.85 (1.29, 2.70) | 1.47 (1.03, 2.15) | 0.001 |
| N-Terminal Pro B-Type Natriuretic Peptide (ng/L) | 12796.00 (5082.00, 35000.00) | 5079.50 (2357.25, 13893.00) | <0.001 |

**Supplementary Table 4 Hyperparameter settings of machine learning.**

| Module | Hyperparameter | Value |
| --- | --- | --- |
| Global Random Seed | random_state | 2025 |
| Cross Validation | KFold.n_splits | 5 |
| Feature Selection | k_features | 2 → 88 (step 2) |
|  | forward | True |
|  | floating | False |
|  | scoring | 'r2' |
|  | cv | KFold(5) |
| RandomForestRegressor | n_estimators | 20 |
|  | max_depth | 5 |
| Main Classifiers (Training Phase) | | |
| LogisticRegression | default | sklearn defaults |
| DecisionTreeClassifier | default | sklearn defaults |
| LinearSVC | default | sklearn defaults |
| MultinomialNB | default | sklearn defaults |
| RandomForestClassifier | n_estimators | 100 |
| XGBClassifier | scale_pos_weight | 2.6 |
